# Microanatomy of the stem-turtle *Pappochelys rosinae* indicates a predominantly fossorial mode of life and clarifies early steps in the evolution of the shell

**DOI:** 10.1038/s41598-019-46762-z

**Published:** 2019-07-18

**Authors:** Rainer R. Schoch, Nicole Klein, Torsten M. Scheyer, Hans-Dieter Sues

**Affiliations:** 1 Staatliches Museum für Naturkunde Stuttgart, Rosenstein 1, D-70191 Stuttgart, Germany; 2 Institut für Geowissenschaften, Abteilung Paläontologie, Nussallee 8, 53115 Bonn, Germany; 3 Universität Zürich, Paläontologisches Institut und Museum, Karl-Schmid-Strasse 4, CH-8006 Zurich, Switzerland; 4 Department of Paleobiology, National Museum of Natural History, Smithsonian Institution, MRC 121, Washington, DC 20560 USA

**Keywords:** Palaeoecology, Palaeontology

## Abstract

Unlike any other tetrapod, turtles form their dorsal bony shell (carapace) not from osteoderms, but by contribution of the ribs and vertebrae that expand into the dermis to form plate-like shell components. Although this was known from embryological studies in extant turtles, important steps in this evolutionary sequence have recently been highlighted by the Triassic taxa *Pappochelys*, *Eorhynchochelys* and *Odontochelys*, and the Permian *Eunotosaurus*. The discovery of *Pappochelys* shed light on the origin of the ventral bony shell (plastron), which formed from enlarged gastralia. A major question is whether the turtle shell evolved in the context of a terrestrial or aquatic environment. Whereas *Odontochelys* was controversially interpreted as aquatic, a terrestrial origin of turtles was proposed based on evidence of fossorial adaptations in *Eunotosaurus*. We report palaeohistological data for *Pappochelys*, a taxon that exemplifies earlier evolutionary stages in the formation of the bony shell than *Odontochelys*. Bone histological evidence reveals (1) evolutionary changes in bone microstructure in ribs and gastralia approaching the turtle condition and (2) evidence for a predominantly amphibious or fossorial mode of life in *Pappochelys*, which support the hypothesis that crucial steps in the evolution of the shell occurred in a terrestrial rather than fully aquatic environment.

## Introduction

**Figure 1:**
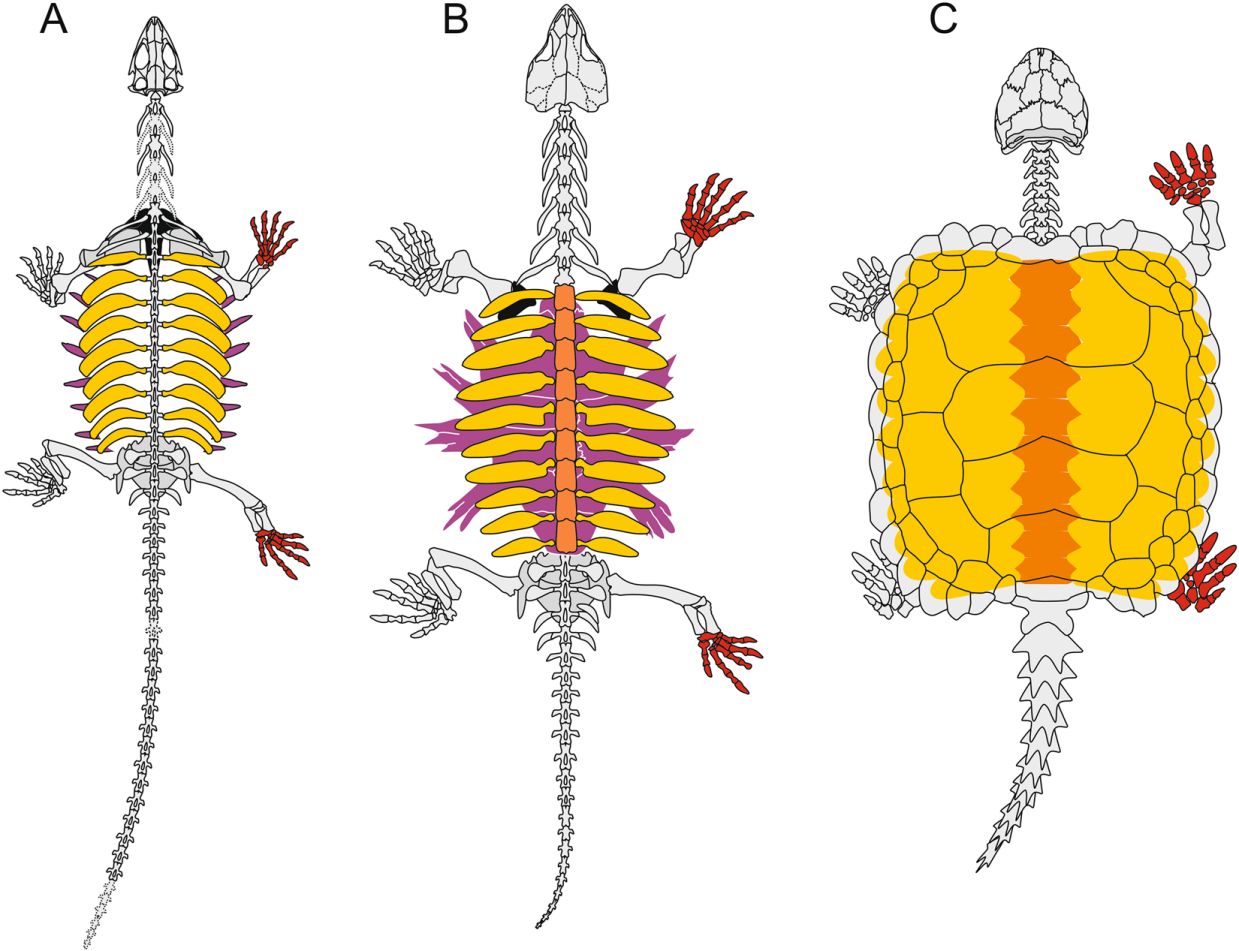
Outline drawings of stem-turtles *Pappochelys rosinae* (**A**), *Odontochelys semitestacea* (**B**), and *Proganochelys quenstedti* (**C**). Modified from Schoch & Sues (2017).

The origin of the turtle shell has remained controversial for centuries. Recent finds of fossil stem-turtles have expanded our knowledge on the origin of the turtle skeleton and in particular the structure of its shell^1–5^. Turtles are unique among tetrapods in the possession of a bony shell that is integrated with the axial skeleton. The dorsal portion of this shell (carapace) is formed by both endoskeletal and exoskeletal components (Fig. 1): broadened ribs and neural spines are combined with secondary metaplastic ossifications, which are sutured to form a rigid shell^6^. Together, they encompass continuous structures reaching from endoskeletal layers well into the dermis^7^. In contrast to other reptiles, the trunk ribs are immobile and extend dorsolaterally into the dermis, where they are covered by dermal bone to form composite elements (costals). By contrast, the ventral portion of the bony shell (plastron) forms without any endoskeletal contribution but its origin has remained an open question until recently^6–11^. It has long been assumed that much of the plastron formed through fusion of the gastralia except for the anterior portion, which comprises ventral bones of the pectoral girdle^7,11^.

The recent discoveries of three stem-turtles, *Odontochelys* and the slightly older *Eorhynchochelys* from the Late Triassic (Carnian) of China^1,2^ and *Pappochelys* from the Middle Triassic (Ladinian) of Germany^3^, have added crucial palaeontological evidence concerning the evolution of the turtle shell. These stem-turtles share broadened, T-shaped ribs (in transverse section) that do not form a complete carapace, but they differ in the formation of the plastron, revealing two steps in the evolutionary sequence of the formation of this structure. Unlike the fully-formed turtle-like plastron of *Odontochelys*^1^, the venter of *Pappochelys* retains a full complement of paired, unusually large and diverse gastralia^3,12^. *Eorhynchochelys* also has gastral ribs but their arrangement is unclear^2^. *Pappochelys*, *Eorhynchochelys*, and *Odontochelys* fill the gap between fully shelled stem-turtles (e.g., *Keuperotesta*^13^, *Proterochersis*^14^, *Proganochelys*^15^) and the oldest known putative stem-turtle *Eunotosaurus*, which has broadened but still very long trunk ribs that are T-shaped but simple paired gastralia^4,8^. The broadened ribs of *Eunotosaurus* and *Odontochelys* have also been a topic in the debate concerning the aquatic vs. terrestrial origin of turtles^8,9^. Although the detailed description of *Pappochelys*^3,12^ revealed many transitional aspects of the osteology between the former mentioned taxa, two major questions in the origin of turtles remain: (a) what was the evolutionary sequence leading to the development of the bony shell in turtles and (b) what was the ancestral mode of life for Pan-Testudines?

As a first step toward addressing these questions, we examined the bone microstructure of *Pappochelys rosinae*. To this end, we have sectioned limb-bones, vertebrae, ribs, and gastralia of this taxon and also employed micro-CT-scanning data. Microanatomy of *Pappochelys* has then been compared with data for other tetrapods, as well as other representatives of the turtle clade. We hypothesise that the evolution of habitat preference among early stem-turtles did not occur in a clean “step‐wise” manner.

## Results

### Limbs

**Figure 2:**
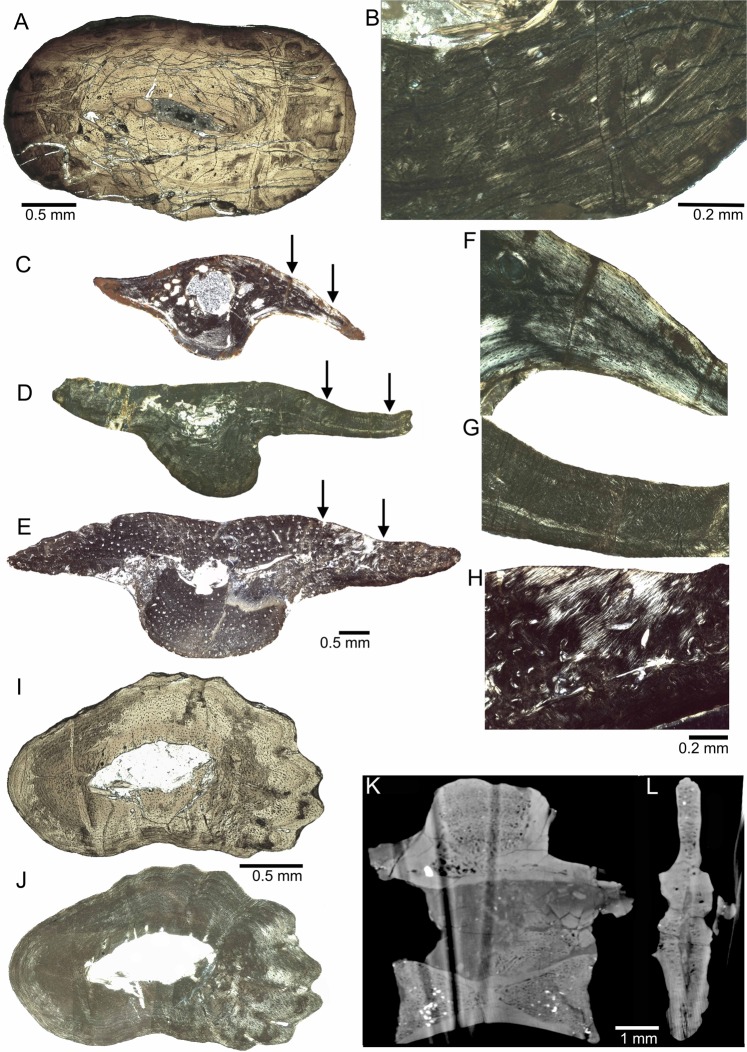
Microanatomy and histology of studied elements of *Pappochelys rosinae*. (**A**) Femur (SMNS 91357) in normal light and (**B**) Detail of the cortex in polarized light. Note the dorsoventral compaction of the femur and the thick, nearly avascular compact cortex. (**C–H**) Dorsal ribs in polarized light. Arrows in (**C–D**) mark areas of enlargement in (**F–H**). (**C,F**) Dorsal rib (SMNS 91968) exhibiting avascular parallel-fibred cortex with no fibres developed. (**D,G**) Dorsal rib (SMNS 91115) with avascular parallel-fibred cortex and short as well as angled fibres in the dorsal flange developed. (**E,H**) Dorsal rib (SMNS 92069) with a high number of longitudinal vascular canals in a parallel-fibred cortex with long fibres in the dorsal flange developed. (**I**) Ornamented gastralium (SMNS 91895) in normal and (**J**) polarized light. Note the long fibres in the dorsal and ventral portion. (**K**) Micro-CT-scan of dorsal vertebra (SMNS 96939) in longitudinal (mediolateral) view, and (**L**) in highly compacted transverse (anteroposterior) view.

Femur and humerus in *Pappochelys rosinae* both display a very small medullary cavity (~3% of the surface ratio) (Fig. 2A,B) surrounded by a thick compact periosteal cortex, revealing osteosclerosis. The central medullary cavity is surrounded by a narrow zone of cancellous bone in the humerus (Suppl. Fig. S1A) but not in the femur (Fig. 2A,B). The parallel-fibred matrix is only poorly vascularized by small longitudinal simple canals. Osteosclerosis solely results from the thickened cortex. Bone compactness of femur SMNS 91357 is 96.8%. The analysis of this femur with Bone Profiler^16^ and including the resulting values of this analysis into the Supplementary Excel Sheet (SOM4) of^17^ revealed parameters that suggests an amphibious mode of life.

When compared to terrestrial amniotes^18^ as well as to some marine sauropterygians^19,20^, the medullary cavity in long bones of *Pappochelys* is rather small. It differs even more from aquatic turtles, which have no clear-cut medullar cavity but spongiosa in the medullar region^18^. Only some placodonts, pistosaurs, and a few large nothosaurs show a similar reduced medullary cavity combined with a thickened cortex^20,21^. However, most aquatic amniotes achieve osteosclerosis by completely different patterns: by incomplete endochondral ossification (i.e., retainment of calcified cartilage) or by intensive endosteal deposits (i.e. filling the medullary region by endosteal bone)^22^.

Microanatomy of long bones of *Pappochelys* differs from that in extant turtles^23,24^ as well as from the microstructure revealed by micro-CT-scans in *Proganochelys* (Suppl. Fig. S1B). In contrast to turtles, femur and humerus of *Pappochelys* lack inner spongiosa but have a tubular structure with an open, although rather small cavity. Such a tubular inner structure is common in most extant terrestrial and semi-aquatic amniotes^25–28^. Interestingly, unlike in many other tetrapods (e.g.^18^), the microanatomy of turtle humeri does not reveal a clear signal reflecting the preferred mode of life of the respective taxa^24^. The cortex of the femur of *Pappochelys* has in general a much lower vascular density compared to turtles.

### Vertebrae

The micro-CT scanned dorsal vertebra was heavily crushed mediolaterally by compaction of the surrounding sediment. The anterior and posterior part of the amphicoelous centrum and the centre and dorsal portion of the neural spine are cancellous (Fig. 2K,L). These cancellous areas have been identified as endochondral territories. In general, the cavities in the endochondral part are small (except for the antero-ventral part of the centre of the neural spine) and connected by thick trabeculae, resulting in a tight network similar to that of terrestrial amniotes^29–32^. The endochondral areas are surrounded by a compact, locally thick, periosteal bone (Fig. 2K,L). However, a distinct separation into an inner bony ring surrounding the neural canal (as described for squamate vertebrae^31,32^) is not visible. In longitudinal view, the neural arch is heavily fragmented (Fig. 2K) but the transversal view (Fig. 2L) reveals compact periosteal tissue. The anterior and posterior endochondral part of the centrum are separated by a pillar of compact periosteal bone. The overall impression of the dorsal vertebra is osteosclerotic.

Amniote vertebrae display a high degree of morphological and microstructural variability, and interpretation of this variation remains difficult^33^. Preliminary analyses testing possible associations between vertebral structure and mode of life suggest that vertebrae of fossorial taxa are denser than those of terrestrial taxa (both sharing a small number of relatively thick trabeculae) and those in aquatic taxa are intermediate in density as expressed by a large number of relatively thin trabeculae^33,34^. This evidence renders an aquatic life style—based on the microstructure of the dorsal vertebra—unlikely for *Pappochelys* but suggests a fossorial life style.

The microstructure of dorsal vertebrae in turtles is difficult to compare due to their morphological changes in the course of shell development. In a previous study of turtle shell bones^35^ a vertebra of the aquatic *Platemys platycephala* (^35^ fig. 31a) and a neural of the terrestrial *Terrapene carolina triunguis* (^35^ fig. 54e) with the corresponding vertebral centrum attached, among others, were figured. Both differ in structure: *Terrapene* has a rather large vertebral canal surrounded by thin trabeculae in thin neural arch pedicels and vertebral centra, whereas the vertebral canal of *Platemys* is also extensive, but surrounded by thick trabeculae in rather stout pedicels and a less reduced centrum. Thus, the vertebral microstructure of both turtles differs from that of *Pappochelys*.

### Ribs

The thoracic ribs of *Pappochelys* form gently curved rods with anterior and posterior processes and a broad but short ventrally expanding ‘bulge’ (Fig. 2C–H)^3,12^. All ribs of *Pappochelys* have an open, round medullary cavity that is lined with a thin layer of endosteal bone. The periosteal matrix consists of parallel-fibred tissue, deposited in varying degrees of organization and which is partially grading into lamellar bone. Rib SMNS 91968 is nearly avascular but shows some large erosion cavities (Fig. 2C,F; Suppl. Fig. S1C). Vascular density is low in SMNS 91115, displaying few longitudinal simple vascular canals (Fig. 2D,F; Suppl. Fig. S1D), whereas rib SMNS 92069 is heavily scattered by numerous longitudinal primary osteons (Fig. 2E,H; Suppl. Fig. S1E). Despite the presence of a medullary cavity the overall impression of the ribs is osteosclerotic, as well (compare to^34,36^).

The upturned and downturned formation of the processes reflect either a slight overlap with the adjacent ribs or at least a musculotendinous connection between adjacent processes (as is the case of broadened ribs in some edentates^37^). The processes are not as broad and widely imbricating as in *Eunotosaurus*, but more closely resemble those of *Odontochelys*, especially in the asymmetric outline of the flanges in ventral view^9^.

In *Pappochelys*, as in *Eunotosaurus*, the cortex lacks interwoven structural fibres or other structures that would indicate metaplastic ossification of dermis. However, incorporation of anchoring fibres is found in the transversal process of two ribs (SMNS 91115, SMNS 92069; Fig. 2D–H) of *Pappochelys*, which may form an early stage in the evolution of metaplastic ossification. The presence of numerous short and angled fibres (SMNS 91115) and locally Sharpey’s fibres (SMNS 92069) in the processes suggest a strong fibrous connection to those of neighbouring ribs. Thus, in *Pappochelys*, the anterior and posterior processes develop as outgrowths of the rib periosteum. In turtles, this connection is present in the sutural margins of the shell bones laterally. The ribs of *Pappochelys* differ from those of *Eunotosaurus* in lacking a woven-fibred portion within the ventral bulge of the T-shaped cross-section (Suppl. Fig. S1C–E), in a distinctly higher compactness, and morphologically they did not overlap as extensively.

The costalia, which make up a large portion of the carapace in turtles, are homologous with the amniote ribs; they combine costal periosteum with an additional layer of metaplastic bone^7^. This is added dorsal to the original rib *anlage* and develops from interwoven structural fibres. *Pappochelys* and extant turtles share a (sub)circular cartilage *anlage* of the rib, which becomes surrounded by a layer of periosteal bone. From this periosteal layer, early outgrowths of bony spiculae grow into the surrounding dermal tissue, as is also the case in *Eunotosaurus*^5^. The vascular cavities surrounding the rib *anlage* are formed in the same way in *Pappochelys* and crown turtles^5^.

Ribs of *Pappochelys* are unique considering their shape, inner structure, and tissue, although some extinct taxa share comparable broadening of their ribs (see^5^).

### Gastralia

The large gastralium SMNS 91895 has a smooth medial margin, whereas the lateral margin is increasingly lobate from the interior to the external bone surface, leading to the formation of several pronounced prongs/ridges interspersed with valleys (Fig. 2I,J). The gastralium has a large, central medullary cavity that roughly matches the shape of the cross-section and is partially lined by a thin layer of endosteal bone. Two more, smaller and not ornamented, gastralia were sectioned along with the rib from SMNS 91115. The larger one (SMNS 91115a) has an open medullary cavity, whereas the smaller one (SMNS 91115b) shows a medullary region that contains endosteal bone. The latter might be related to a more distal sampling location. The low vascularized (SMNS 91895) and avascular (SMNS 91115a, b) cortices of gastralia are made of parallel-fibred matrix. The cortex of the large ornamented gastralium SMNS 91895 contains numerous large, globular osteocytes, whereas osteocytes are far less numerous and flat in the other two gastralia. The lateral portion of the ornamented large gastralium (SMNS 91895) contains numerous prominent Sharpey’s fibres and shorter fibres in the inner and outer cortex, whereas the medial portion shows no distinct fibres (Fig. 2J). The shorter fibres might also have anchored soft-tissue to the bone. SMNS 91115a and b, both lack any kind of fibres.

Gastralia of *Pappochelys* are superficially pachyostotic^3,12^, but retain a hollow internal structure, which make them less osteosclerotic when compared to aquatic amniotes. In plesiosaurs, gastralia have a cancellous internal structure^38^ whereas those of the ichthyosaur *Mixosaurus*^39^, eosauropterygians^34^, and the rhynchocephalian *Palaeopleurosaurus*^40^ are compact without any internal spaces. Instead, gastralia of present-day alligators show diffuse mineralisation^41,42^ and another gastralium has a small cancellous centre^43^. The gastralia of *Pappochelys* with open medullary cavities more closely resemble those of terrestrial taxa, such as two paracrocodylomorph archosaurs from the same deposit (*Batrachotomus*^44^ and a yet undescribed small rauisuchian).

The presence of a large central cavity, the development and density of fibres and the ornamented margin in large gastralia of *Pappochelys* are unique among tetrapod gastralia reported to date.

## Discussion

### Life style

Morphology and microanatomy do not always correlate directly to habitat preference in many species, as aquatic and terrestrial species often share similar histological and morphological features owing to frequent evolutionary reversals in habitat preference^33,34^. This is especially true for turtles^24^. In *Pappochelys*, the histology and microanatomy of limb-bones, vertebrae, ribs and gastralia reveals a complex picture, which is in this combination—for each bone as well as in sum—unique. Although all bones of *Pappochelys* are osteosclerotic, microanatomical patterns and processes involved differ from that of what is known for aquatic amniotes and a clear identification of life style for *Pappochelys* is hampered.

However, the simple presence of an increase in cortex thickness accompanied by a reduction in medullary cavity size need not indicate aquatic dispositions: the same features have been reported for the terrestrial lepidosaur *Sceloporus*^30^ as well as the burrowing potential stem-turtle *Eunotosaurus*^8^. In combination with numerous osteological correlates^3,12^, we argue (on the basis of the microanatomy of the vertebra) that a terrestrial (i.e. fossorial) or modest amphibious mode of life (based on the analysis of the femur with bone profiler) is much more plausible for this taxon than a fully aquatic one.

### Turtle shell development

**Figure 3:**
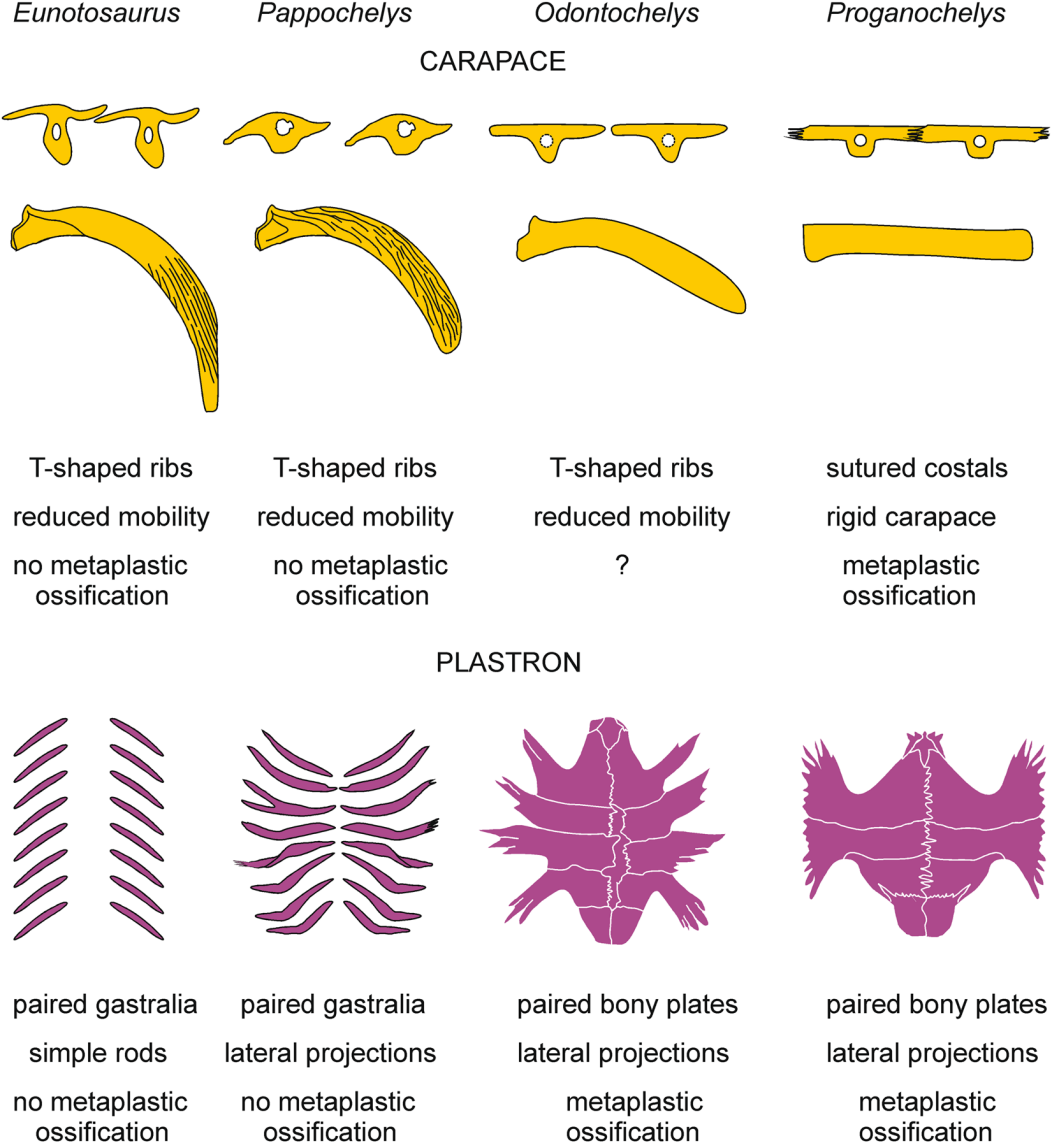
Proposed sequence of structural changes to the carapace and plastron in *Eunotosaurus africanus* and the Triassic stem-turtles *Pappochelys rosinae*, *Odontochelys semitestacea*, and *Proganochelys quenstedti*.

**Figure 4:**
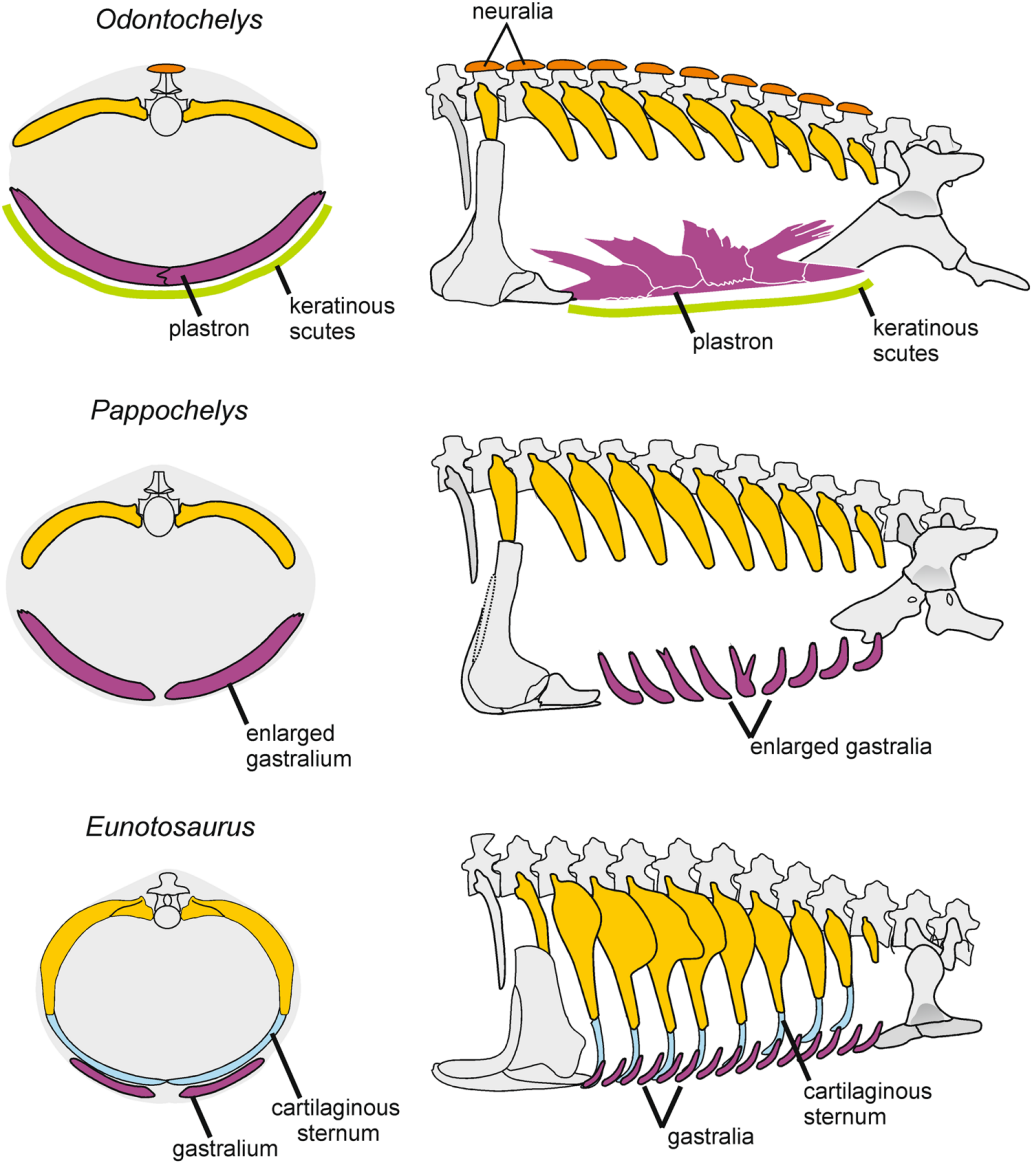
Proposed hypothetical scenario for the evolution of the carapace and plastron in *Eunotosaurus africanus* and the Triassic stem-turtles *Pappochelys rosinae* and *Odontochelys semitestacea*. Left, cross-section of mid-trunk; right, trunk in lateral view.

The unique histology (i.e. presence of fibres) of the ribs and gastralia combined with their specialized morphology^3,12^ gives insights into the development of the turtle carapace and plastron. *Pappochelys* thus exemplifies an important step in the evolution of the turtle shell (Figs 3 and 4). This is because its short and broadened ribs were already confined to the dorsal part of the trunk and located in a superficial position to extend well into the dermis. The dorsal surface of the ribs was heavily ornamented, consistent with a shallow position within the dermis but also indicating that they were not yet covered by keratinous scutes. Ventrally, the gastralia were greatly thickened to form a rigid basket in *Pappochelys*. The ribs were still somewhat moveable, as indicated by the joints as well as the abundance of fibres in the horizontal ‘wings’ of the ribs. These fibres suggest that strain acted on them, presumably from musculature ventilating the lungs. At the same time, other fibres indicate that successive ribs were already interconnected to form a protocarapace. This is consistent with the curved, wave-like cross-section of the ribs, which indicates slightly imbricating flanges. The articulation of free ribs might explain why the carapace evolved more slowly than the plastron along the turtle stem, as some mobility of the rib cage continued to be required for respiration.

The incipient dorsal shell in *Eunotosaurus* (Fig. 4) was recently studied in detail^5^. It consisted of greatly expanded thoracic ribs that had started to expand into the dermis. As in other amniotes, these ribs were strongly curved and elongate, probably still associated with a cartilaginous sternum ventrally. *Pappochelys* may well have elaborated on such an early stage by separating the ribs from the sternum and adding enlarged gastralia. In contrast to *Eunotosaurus*, the ribs of *Pappochelys* are shorter and less ventrolaterally curved (Fig. 4). Recently, the suppression of the sternum in early development has been demonstrated to be an essential prerequisite for the formation of the turtle plastron – this involves reprogramming of cartilage-producing chondroblasts into bone-forming osteoblasts^10^.

The transformation of modified gastralia into part of the turtle plastron is of particular interest. *Eunotosaurus* had thin rod-like gastralia much as in most amniotes, but they were reduced to two elements per transverse row, lacking the medial element present in most reptiles^5^. *Pappochelys* also has two gastralia per row but the individual elements are much larger than in *Eunotosaurus* and regionally differentiated^12^. They are heavily ornamented with ridges on the ventral side and some are twisted, indicating a more complex three-dimensional arrangement than in the primitive amniote condition^9^. Unlike many other reptiles, *Pappochelys* has only one row of gastralia per vertebral segment. The finger-like projections at the distal ends of these gastralia closely resemble the distal bifurcations and projections on the plastral elements of *Odontochelys*^3^.

Most gastralia of *Pappochelys* bear parallel ridges, especially along their ventral surfaces. These ridges merge into finger-like projections at the distal ends of some gastralia^12^. Like the intensity of ornamentation, the presence and density of fibres in gastralia of *Pappochelys* are unique among tetrapod gastralia reported to date. They probably indicate an expansion of gastralia from the layer of abdominal musculature well into the dermis. Although of different embryological origin, the gastralia thus parallel the tendency of ribs to expand into layers of the dermis, which were first steps toward the formation of carapace and plastron.

How did these enlarged gastralia transform into the plastron? In *Pappochelys*, fusion of neighbouring gastralia has not been confirmed by bone histology, because even the broadest elements with notable bifurcations have single medullary cavities. Instead, single gastralia appear to have split at various levels, starting from distal levels near the tips up to about midlength of the element. Hence, the next step in the evolution of the plastron may have involved (1) large-scale fusion of adjacent gastralia to form plates (as is indicated by early development of extant turtles^11^ and (2) metaplastically ossifying preformed dermal tissue around the gastralia. The latter occurs mainly during posthatching development in the plastron formation of both, extant hard-shelled and soft-shelled turtles, as has been shown by shell bone histology^35,45,46^. The evolutionary sequence and timing leading to a fully ossified plastron, however, can only be resolved by future discoveries of taxa intermediate between *Pappochelys* (and *Eorhynchochelys*) and *Odontochelys* or by examination of the bone microstructure of the plastral elements in *Odontochelys*. Although occupying an intermediate position between *Pappochelys* and *Odontochelys*^2^, *Eorhynchochelys* does not add evidence here, because only few dislocated gastral ribs are visible between the dorsal ribs.

The crucial question is whether *Pappochelys* evolved these new shell features within the same functional context as *Eunotosaurus*, namely a fossorial lifestyle, or whether it was an aquatic animal that used its ‘proto-shell’ for protection against predators or as skeletal ballast to remain submerged.

Unlike *Eunotosaurus*, which is found in floodplain deposits together with other probably burrowing tetrapods^8^, *Pappochelys* was discovered in mudstones that formed in a small freshwater lake^47^, whereas the more derived *Eorhynchochelys* and *Odontochelys* occur in shallow marine strata^1,2^. What do these occurrences tell us about the setting in which the turtle shell evolved?

Recently, the analysis of skeletal features in *Eunotosaurus* indicated that this taxon shares various traits with fossorial amniotes^8^. It was argued that the broadened and imbricating dorsal ribs provided rigidity during digging/burrowing, as did the foreshortened trunk. Numerous features in the limbs fit this interpretation, such as the robust humerus and ulna, the short manus and pes with long and robust claws suited for powerful digging. For *Eorhynchochelys* an amphibious lifestyle in near shore-terrestrial habits, as well as digging activity based on robust limb morphology and enlarged terminal phalanges was also hypothesised^2^. This raises the question whether *Pappochelys* shows similar features, or which correlates can be found in that taxon regarding its lifestyle. The grade formed by *Pappochelys, Eorhynchochelys* and *Odontochelys* is especially interesting, because the evolution of the plastron has been considered to have taken place in the water^1,48^.

Except for the osteosclerotic long bones and vertebra, *Pappochelys*, like *Odontochelys*, presents no apparent morphological correlates of an aquatic mode of life, in contrast to the evidence in similar-sized marine diapsids such as *Neusticosaurus* and *Claudiosaurus*^18^. Especially the pachypleurosaurs *Neusticosaurus* and *Serpianosaurus* share a wide range of aquatic adaptations with their larger sauropterygian relatives (placodonts, eusauropterygians): (1) development of pachyostosis and osteosclerosis in the limbs, ribs, vertebrae and gastralia, (2) lack of ossification of the carpals and tarsals, (3) ventral expansion of the limb girdles, (4) laterally compressed tail, (5) flattening of fore- and hind limbs^18^. Except for osteosclerotic long bones and vertebra and pachyostotic ribs and gastralia none of these features is present in *Pappochelys* and (apart from the as yet undocumented bone histology) *Odontochelys*. Likewise, the spongiose medullar region, a feature of extant aquatic turtles^18^, is absent in *Pappochelys*.

Furthermore, consistent with *Eunotosaurus*, the short manus and pes in *Pappochelys*, *Eorhynchochelys* and *Odontochelys* are found also in fossorial taxa, and the long and robust unguals suggest a mode of life that involves digging^8^.

The occurrence of *Pappochelys, Eorhynchochelys* and *Odontochelys* in lake or shallow marine sediments does not imply a fully aquatic lifestyle. At Vellberg, the type locality of *Pappochelys*, terrestrial taxa were found in large numbers together with remains of aquatic taxa, indicating that land-dwelling forms were easily washed in, or terrestrial taxa were preserved during episodes of drought, for which sedimentological evidence has been presented^47^. *Pappochelys* is a rather common reptile in that lake deposit, but the skeletons are usually heavily affected by predation (most specimens forming regurgitates and coprolites of larger predators). Further evidence is provided by the autecology of *Pappochelys*: the skeleton of the holotype of *P. rosinae* contains bones of two tiny reptiles, which are juveniles of a small diapsid that is similar to the Early Triassic lepidosauromorph *Sophineta*, a terrestrial taxon^49^.

The available morphological and microanatomical evidence indicates that *Pappochelys* was not a fully or predominantly aquatic taxon. Instead, some features identified in *Eunotosaurus* as indicative of fossorial habits, such as the broadened ribs and robust claws, are also present in *Pappochelys* and *Odontochelys*. In addition, the microanatomical structure of the dorsal vertebra in *Pappochelys* points to a fossorial life style as well. The feature potentially indicating an amphibious lifestyle, the osteosclerosis present in all studied bones, but especially in the long bones, is equivocal, because it is also found in fossorially adapted terrestrial taxa^8,30^. The enlarged but hollow gastralia of *Pappochelys*, which very unusually exceed the length of the ribs, are likely to have formed a basket that gave additional rigidity to the trunk, which would have been important during digging. They might have compensated for the reduced role of the thoracic ribs, which were proportionately shorter than in most amniotes and could not strengthen the flanks.

*Pappochelys* may have preferred riparian habitats, as suggested by its abundance in the Vellberg lake deposit. It evidently fed on terrestrial tetrapods and fell prey to larger amphibious or aquatic predators. Based on the currently available data, the early phases in the evolution of the turtle shell took place in a terrestrial rather than aquatic setting, and the driving selective forces were likely not protection in an aquatic environment but rather functional demands to strengthen the trunk during digging.

### Material

All material of *Pappochelys rosinae* included in this study (Table 1) was recovered from the type locality (Schumann quarry, Eschenau, Vellberg municipality, Baden-Württemberg, Germany) and type horizon (Lower Keuper [Erfurt Formation]; Middle Triassic: Ladinian: Longobardian)^3^. The specimens are housed in the Staatliches Museum für Naturkunde Stuttgart, Germany (SMNS). The sectioned bones are throughout highly diagnostic for *Pappochelys* and were mostly sampled from partial skeletons^12^.

**Table 1:** Thin sectioned and micro-Ct-scanned bones of *Pappochelys rosinae* from the Middle Triassic (late Ladinian) of southern Germany (Eschenau/Vellberg).

Bone	Collection number	Sample	Bone compactness
humerus	SMNS 92084	micro-Ct-scan	97.4%
humerus	SMNS 91013	micro-Ct-scan	98%
femur	SMNS 91357	thin section	96.8%
femur	SMNS 92085	micro-Ct-scan	98.3%
femur	SMNS 91711	micro-Ct-scan	98.7%
dorsal vertebra	SMNS 96939	micro-Ct-scan	~75%/
dorsal rib	SMNS 91968	thin section	88%
dorsal rib	SMNS 92069	thin section	91.2%
dorsal rib	SMNS 91115	thin section	95.2%
ornamented gastralium	SMNS 91895	thin section	89.7%
gastralium	SMNS 91115a	thin section	86.6%
gastralium	SMNS 91115b	thin section	99%

The bone compactness of the dorsal vertebra is an average value combined from the longitudinal as well as from the transversal section. However, it can only be a rough estimate due to the fragmented nature of the neural arch.

Two humeri (SMNS 92084, SMNS 91013), two femora (SMNS 91013, 92085), and one dorsal vertebra (SMNS 96939) have been micro-CT-scanned. The quality of the scans varied due to different levels of infiltration by pyrite in the bones. Thin-sections of one femur (SMNS 91357), two dorsal ribs (SMNS 91115, SMNS 91968), and three gastralia (SMNS 91895, SMNS 91115a, b) were produced. The samples came from different individuals except for the rib and two gastralia that were taken from specimen SMNS 91115. Samples of long bones and ribs were taken at mid-shaft.

For microanatomical comparisons, two femora (SMNS 16980, SMNS 17203) of the stem-turtle *Proganochelys quenstedti* from the Late Triassic (Norian) of Trossingen (Germany) were micro-CT-scanned.

## Methods

The thin-sections were produced following standard petrographic methods (Klein and Sander)^50^ and then studied and photographed with a Leica® DM 750 P compound polarizing microscope equipped with a digital Leica® ICC50HD camera. Histological terminology follows Francillon-Vieillot *et al*.^51^. Some samples were micro-CT-scanned with a v|tome|xs by GE phoenix|x-ray at the Steinmann Institut für Geologie, Mineralogie und Paläontologie (StIPB) in Bonn (Germany). Image visualization was performed using VGStudio MAX 2.0 software (Volume Graphics GmbH) and Adobe Photoshop. Cross-sections of samples were transformed into black (bone) and white (cavities and vascular spaces) images to measure bone compactness with a custom-designed pixel-counting computer program developed by P. Göddertz (StIPB).

## Supplementary information

Supplement Figure and Legend

## Data Availability

All data generated or analysed during this study are included in this published article (and its Supplementary Information Files).
