# Supplementary material for: Microanatomy of the stem-turtle Pappochelys rosinae indicates a predominantly fossorial mode of life and clarifies early steps in the evolution of the shell
Source: Sci Rep. 2019 Jul 18;9:10430. doi: 10.1038/s41598-019-46762-z (PMC6639533; doi:10.1038/s41598-019-46762-z)
Supplement: Supplementary file 1 — Supplement Figure and Legend [file 41598_2019_46762_MOESM1_ESM.pdf]

Microanatomy of the stem-turtle *Pappochelys rosinae* indicates a predominantly fossorial mode of life and clarifies early steps in the evolution of the shell

Rainer R. Schoch[1,*], Nicole Klein[1,2], Torsten M. Scheyer[3,*] & Hans-Dieter Sues[4]

[1]Staatliches Museum für Naturkunde Stuttgart, Rosenstein 1, D-70191 Stuttgart, Germany.
[2]Institut für Geowissenschaften, Abteilung Paläontologie, Nussallee 8, 53115 Bonn, Germany.
[3]Universität Zürich, Paläontologisches Institut und Museum, Karl-Schmid-Strasse 4, CH-8006 Zurich, Switzerland.
[4]Department of Paleobiology, National Museum of Natural History, Smithsonian Institution, MRC 121, Washington, DC 20560, U.S.A.
* Corresponding authors: rainer.schoch@smns-bw.de; tscheyer@pim.uzh.ch

Supplementary Figure S1

A, Midshaft cross section of humerus of *Pappochelys rosinae* (SMNS 92084) based on micro-CT-data. Note the poor resolution, which might be due to pyrite minerals incorporated into the bone during diagenesis.
B, Midshaft cross section of femur of *Proganochelys* (SMNS 16980) based on micro-CT-data.
C-E, Detail of periosteal cortex in the ventral bulge in dorsal ribs of *Pappochelys rosinae*.
C, Avascular highly organized parallel-fibred tissue in SMNS 91968. The angled osteocytes might indicate a somewhat angled section plane.
D, Low vascular density in parallel-fibred tissue scattered by simple longitudinal vascular canals in SMNS 91115.
E, High vascular density in low organized parallel-fibred tissue interspersed by longitudinal primary osteons in SMNS 92068.

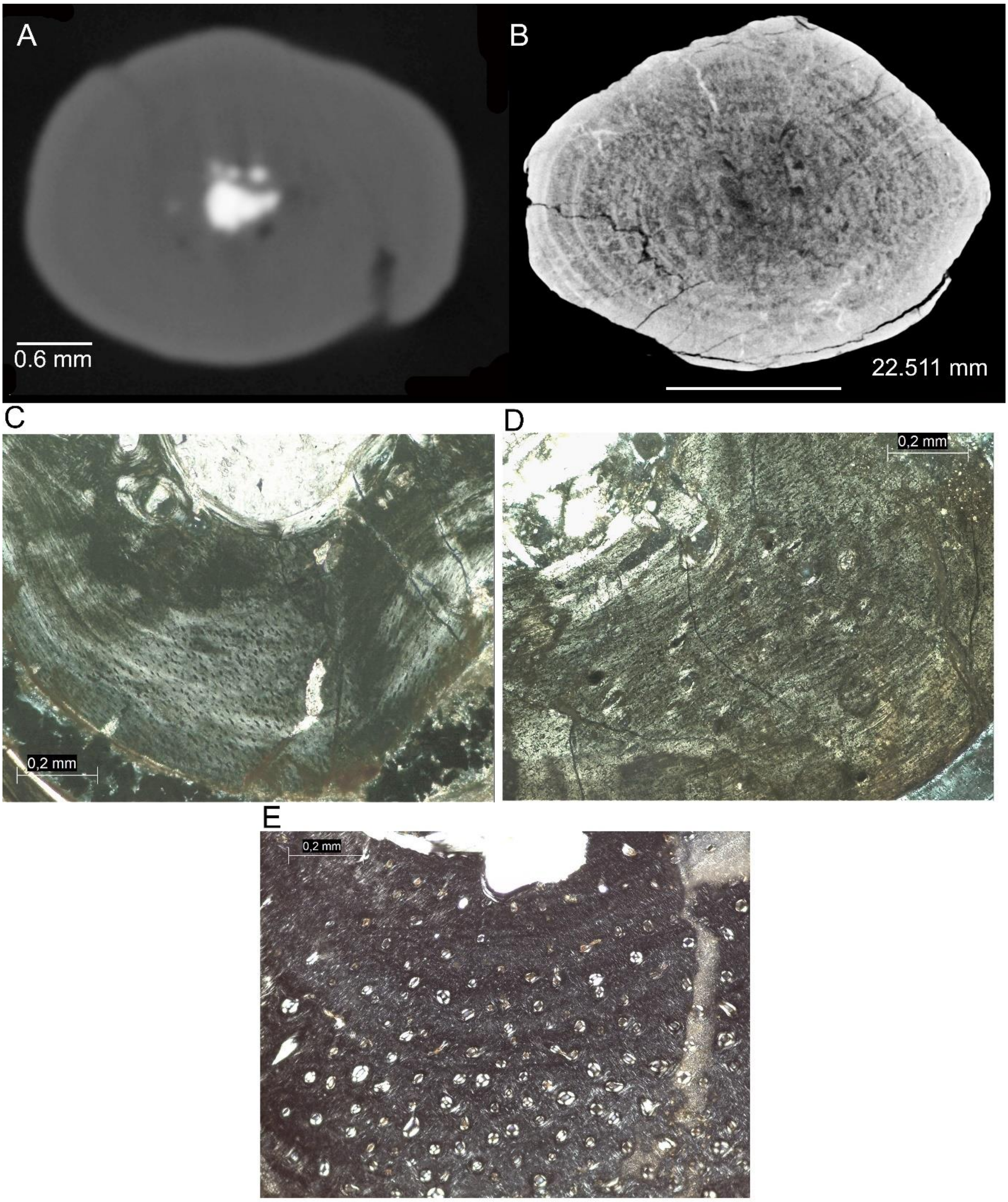
A
0.6 mm
B
22.511 mm
C
0,2 mm
D
0,2 mm
E
0,2 mm
